# An implantable electrical stimulator used for peripheral nerve rehabilitation in rats

**DOI:** 10.3892/etm.2013.1110

**Published:** 2013-05-13

**Authors:** BIYU RUI, SHANGCHUN GUO, BINGFANG ZENG, JINGWU WANG, XIN CHEN

**Affiliations:** 1 Department of Orthopaedics, Shanghai Sixth People’s Hospital, Shanghai Jiaotong University, Shanghai 200233;; 2 Department of Orthopaedics, Shanghai Ninth People’s Hospital, Shanghai Jiaotong University, Shanghai 200011, P.R. China

**Keywords:** implantable stimulator, animal model, electrical stimulation, peripheral nerve injury

## Abstract

This study evaluated an implantable electrical stimulator using a sciatic nerve injury animal model, and ethological, electrophysiological and histological assessments. Forty Sprague-Dawley rats were used in the study, and were subjected to crushing of the right sciatic nerve with a micro-vessel clamp. Electrical stimulators were implanted in twenty of the rats (the implantation group), while the remaining twenty rats were assigned to the control group. At three and six weeks following the surgery, the sciatic nerve function index (SFI) and the motor nerve conduction velocity (MCV) were demonstrated to be significantly higher in the implantation group compared with the control group (P<0.05). Histological analysis, using hematoxylin and eosin (H&E) staining, showed the typical pathological atrophy, and an assessment of the nerve that had been crushed revealed distal axonal breakdown in the control group. These results suggest that the implantable electrical stimulator was effective, and was suitable for implantation in a Sprague-Dawley rat model.

## Introduction

Peripheral nerve injury is a frequent and severe complication following orthopaedic trauma. The treatment of peripheral nerve injury remains a challenge, despite the focus that has been placed on it, as none of the traditional therapies have been demonstrated to produce entirely satisfactory results. One of the potential methods for improving nerve regeneration and the restoration of function is electrical stimulation.

Studies have indicated that electrical stimulation may promote the speed and accuracy of motor and sensory axon regeneration (1–4). Therefore, there have been investigations into the use of functional electrical stimulation (FES) in the induction of peripheral nerve regeneration. The results of *in vitro* (5,6) and *in vivo* (7,8) studies have revealed that a weak electric field enhances neurite outgrowth. Numerous investigations have been performed in this field (1–4,9), and the histomorphometric and electrophysiological analyses have demonstrated that electrical stimulation may be used to accelerate the maturity of regenerated nerves (1–4,7,8).

The expansion of electronic technologies has led to the rapid development of a number of implantable microsystems that have been used in the treatment of a variety of diseases, including deafness (10,11), arrhythmia (12), plegia (13) and Parkinson’s disease (14). However, there has been limited investigation into the use of implantable electrical stimulators in the treatment of peripheral nerve injuries.

In the current study, we designed an implantable electrical stimulator with suitable parameters, and evaluated the efficacy of the stimulator using an animal model.

## Materials and methods

### 

#### Implantable electrical stimulator design

**Figure 1.**
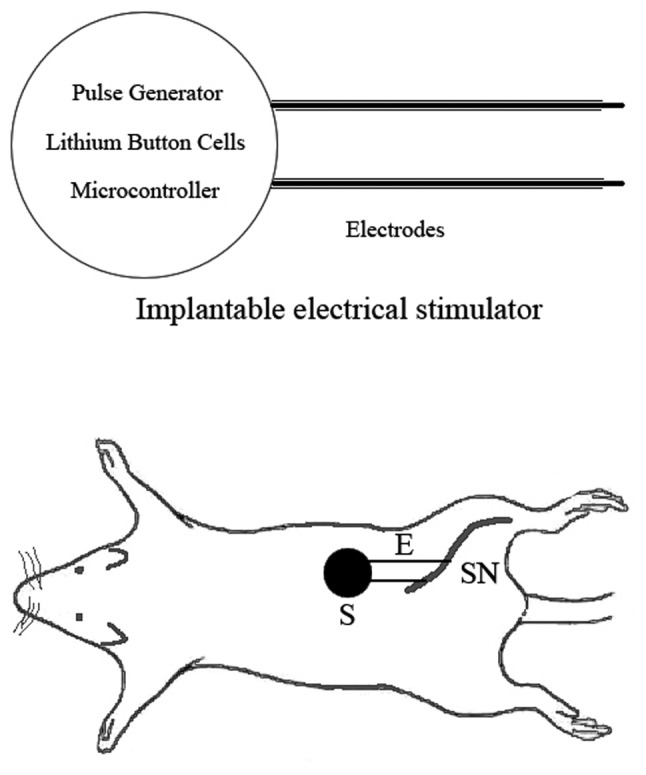
System block diagram of the implantable electrical stimulator. S, implantable electrical stimulator; E: electrodes; SN: sciatic nerve.

**Figure 2.**
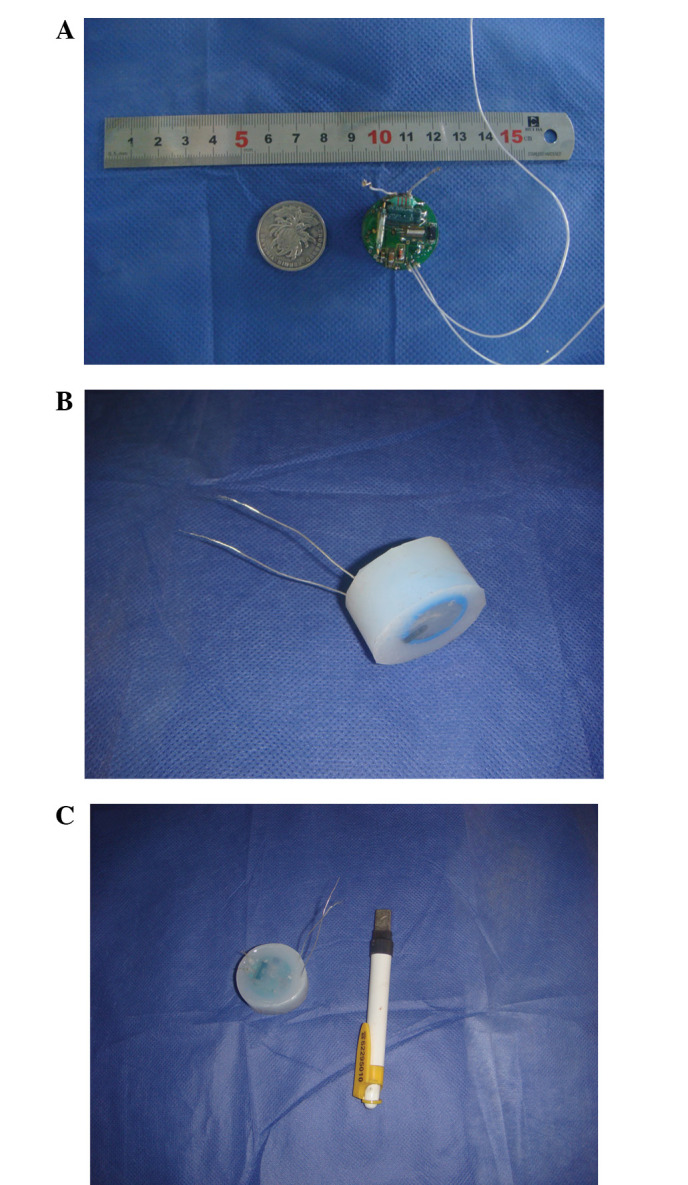
Photographs of the implantable electrical stimulator and electrodes. (A) Overview of the implantable electrical stimulator. The stimulator is the same size as a coin. (B) The implantable electrical stimulator following encapsulation. (C) The stimulator and the external magnetic switch.

A system block diagram of the implantable electrical stimulator is displayed in Fig. 1. The stimulator was designed to be implanted in the backs of the rats, and was created with batteries, bipolar electrodes and integrated circuit (IC) chips, including a micro-controller and a pulse generator chip. The batteries, electrodes and ICs were integrated into the system. The output pads of the stimulator chip were connected to the electrodes, and the power pins were connected to the battery. To supply power to the chip, a 3 V CR2450 lithium button cell battery was used (Shenzhen Eunicell Battery Co., Ltd., Shenzhen, China), which made it possible to operate the device for >8 weeks. The activity of the stimulator was controlled by an external magnetic switch. To protect the stimulator from bodily fluids and mechanical forces, the IC chips, batteries and electrode connector were cast in a medical grade epoxy resin. Following this, the assembled stimulator was coated with a silicone elastomer, and the device was sealed in a gas-permeable bag for ethylene oxide sterilization. Subsequent to the encapsulation, the stimulator measured 30 mm in diameter and 22 mm in depth and weighed 25 g (Fig. 2). The stimulation parameters were selected using the results of our preliminary experiments, which indicated that a stimulation pattern of bipolar pulses with a duration of 400 *μ*sec per phase, an amplitude of 0.8 V and a frequency of 60 Hz was most effective with regard to peripheral nerve rehabilitation in rats (unpublished data).

#### Animal models and surgical procedures for the implantation of the electrical stimulator

All animal experiments were performed in accordance with the Guide for the Care and Use of Laboratory Animals (15). The procedures in the study were designed to minimize the pain or discomfort of the animals, in accordance with the current protocols approved by the Laboratory Animal Ethics Committee of Shanghai Jiaotong University (Shanghai, China). A cohort of 40 healthy 8-week-old Sprague-Dawley rats (weight range, 280–355 g) was recruited for the study. The rats were randomly divided into two groups: implantation (n=20) and control (n=20), and each group was then further divided into two subgroups, according to different time points in the experiment (three and six weeks).

The animals were anesthetized with an injection of 10% chloral hydrate (0.3 ml/100g; Shanghai Chemical Reagent Co., Shanghai, China) into the abdominal cavity. During the surgery, each animal was placed in a prone position, and the skin was prepared. A standard longitudinal incision was made in the right gluteal region, and then the tissue between the subcutaneous and muscular layers was dissected. Following this, the main stem of right sciatic nerve was isolated at mid-thigh level, and was subsequently crushed for 5 min, using micro-vessel clamps with a strength of 2.0 kg. A 1-0 nylon stitch was sutured to the tissue adjacent to the crush site as a marker. In the implantation group, the implantable electrical stimulator was inserted in the back through the subcutaneous tunnel. The silicone elastomer, incorporated during the encapsulation process, was sutured to the subcutaneous tissue to prevent device migration. The two electrodes were wrapped and sutured to the epineurium proximal and distal to the crushed sciatic nerve, respectively, prior to the suturing of the skin incision. In the control group, no measures were taken subsequent to the crushing of the sciatic nerve. Following the surgery, the animals were allowed access to food and water *ad libitum*, in isolator cages that were maintained under a 12 h light-dark cycle at 25°C. Stimulation was performed for 120 min per day in the implantation group. The animals were checked daily, and the stimulation was observed to be well-tolerated in every case.

### Experimental materials

#### Ethological evaluation

Ethological methods were used to observe, record and analyze the behavior of the rats, in terms of the signs and extent of muscular atrophy in the hind limb, gait and cutaneous ulceration, at each time point.

#### Sciatic nerve function index (SFI)

Three and six weeks following the surgery, the SFI was calculated using the method described by Reynolds and Weiss (16). The hind legs of the rats were dyed with ink, to enable the footprints of the healthy (N) and wounded (E) feet to be measured when the rats walked across a piece of white paper. The measurements were taken in three indices, as follows: length of footprint (IPL, from toe to heel), width of toes (ITS, from the 1st to the 5th toe) and width of the middle toes (IIT, from the 2nd to the 4th toe). The results were accurate to 0.1 mm. The SFI was calculated in accordance with the formula described by Bain *et al* (17): SFI=−38.3 [(EPL-NPL)/NPL] + 109.5 [(ETS-NTS)/NTS] + 13.3 [(EIT-NIT)/NIT] −8.8, where EPL is the length of footprint (from toe to heel) of wounded feet, NPL is the length of footprint (from toe to heel) of healthy feet, ETS is the width of toes (from the 1st to the 5th toe) of wounded feet, NTS is the width of toes (from the 1st to the 5th toe) of healthy feet, EIT is the width of middle toes (from the 2nd to the 4th toe) of wounded feet and NIT is the width of middle toes (from the 2nd to the 4th toe) of healthy feet. An SFI value of between 0 and 11% represented normal nerve function, whereas −100% represented complete damage of nerve function, and between −11 and −100% repesented incomplete nerve function recovery.

#### Electrophysiological assessment

Three and six weeks following the surgery, the sciatic nerve at the surgical site was exposed under anesthesia. The proximal site of the crushed section was linked with an electrode, and the gastrocnemius was connected to a recording electrode, in order to deter mine the mean conductive velocity (MCV) of the sciatic nerve.

#### Macroscopic evaluation

The results were evaluated at three and six weeks following the surgery, respectively, with 10 rats from each group assessed at each time point. During the evaluation, the shape, adhesion between the stimulator and the surrounding tissue, the corrosion of the electrodes and neuroma formation at the crush site were observed.

#### Morphology and morphometry

Sections were cut from the gastrocnemius muscles of the rats. Two sections, from the experimental and the contralateral control muscles, respectively, were placed on each slide. The sections were stained using haematoxylin and eosin (H&E). The H&E-stained sections were overlaid by a transparent grid of 1×1 mm squares, in order to measure the complete cross-sectional area (CSA). Ten evenly spaced 1 mm^2^ fields were selected for the microscopic analysis, with the convention that the fibers intersecting the upper and left boundaries were included, whereas those intersecting the lower and right boundaries were excluded. The total number of muscle fibers in the muscle was estimated as the product of the total muscle CSA and the mean number of fibers per square millimeter. Representative digital photomicrographs of H&E-stained sections were taken with an adapted camera (Leica QWin V2.6; Leica Microsystems, Wetzlar, Germany).

#### Transmission electron microscopy

Specimens were obtained from the sciatic nerve at the experimental or the equivalent normal site of the rats. These were fixed in 3.5% glutaraldehyde in 0.1 mol/l sodium cacodylate buffer (pH 7.2) for 2 h at room temperature, and then stored at 4°C in the same solution. Small bundles of fibers were postfixed for 1 h in 1% osmium tetroxide in the same buffer, dehydrated through a graded ethanol series followed by acetone, and then embedded in epoxy resin. Ultrathin sections (30–40 nm) were cut using a Leica Ultracut R microtome (Leica Microsystems) and stained with 4% uranyl acetate and lead citrate. The sections were subsequently examined with a Hitachi H-600 electron microscope (Hitachi, Tokyo, Japan), and representative photomicrographs were taken with an adapted camera.

#### Statistical analysis

The results are expressed as the mean ± standard deviation (SD). The electrophysiological and morphological properties of the control (non-stimulated and contralateral) and stimulated hind limbs were compared using one-way analysis of variance (ANOVA), followed by the Tukey-Kramer post-test, using SPSS software, version 16.0 (SPSS, Inc., Chicago, IL, USA). P<0.05 was considered to indicate a statistically significant difference.

## Results

### 

#### Ethology

All the rats were noted to have normal hind limb muscles, skin and gait prior to the crushing of the sciatic nerve. Three weeks following the surgery, the rats in the control group (n=10) displayed muscular atrophy in the hind limb and marginal gait changes, and at the six-week time point, the control rats demonstrated extensive hind limb muscular atrophy, and marked gait changes. In comparison, at three weeks after surgery, the rats in the implantation group (n=10) presented with atrophic muscles in the hind limb, and minor gait changes. At six weeks, the rats with the implanted electrical stimulator demonstrated marginal atrophy in the muscles of the hind limb, and normal gait.

#### SFI

**Figure 3.**
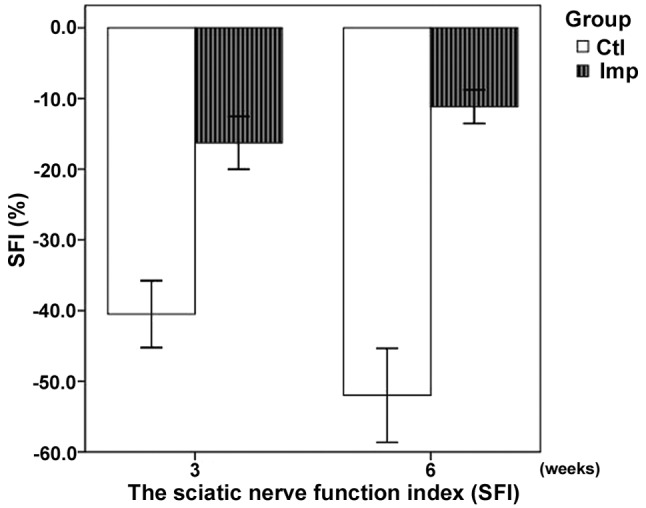
Differences in the sciatic nerve function index (SFI) between the control (Ctl) and implantation (Imp) groups.

The results of the SFI assessment are summarized in Table I. The SFI was significantly higher in the implantation group compared with the control group, at the three- and six-week time points (P<0.05). The SFI demonstrated an improved recovery in the implantation group (Fig. 3).

#### Electrophysiological changes

**Figure 4.**
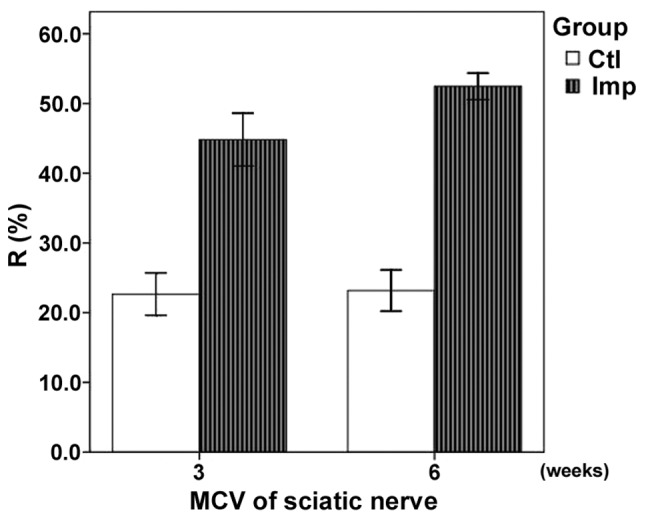
Electrophysiological differences between the sciatic nerves in the control (Ctl) and implantation (Imp) groups. R, recovery rate; MCV, motor nerve conduction velocity.

The results of the electro-physiological assessment are summarized in Table II. The recovery rate (R) of the MCV (R=MCV of the experimental side/MCV of the contralateral side) of the sciatic nerve in the implantation group was significantly higher compared with that of the control group at three weeks (P<0.05). In addition, a significant difference was observed between the Rs of the sciatic nerve MCVs in the implantation and control groups at six weeks (P<0.05). The MCV demonstrated an improved recovery in the implantation group (Fig. 4).

#### Macroscopic evaluation

With regard to the implantation group at three weeks, the electrical stimulator remained in its original size and shape, although the electrodes had adhered to the surrounding tissue, and no neuromas were present at the site where the sciatic nerve had been crushed. Compared with the normal side, the sciatic nerve expressed a thicker epineurium on the surface of the site of the crush injury, and the muscles were marginally paler. At six weeks, no inflamatory reactions were observed in the tissues surrounding the stimulator, and the adhesion of the electrodes was reduced. There were no notable differences between the muscles of the two sides. In the control group, there was extensive degeneration at the site where the sciatic nerve had been crushed, and there were no regenerated axons crossing over the site of the crush injury, or neuroma formation in the proximal nerve. The muscles appreared paler than those from the normal side.

#### Morphology and morphometry

**Figure 5.**
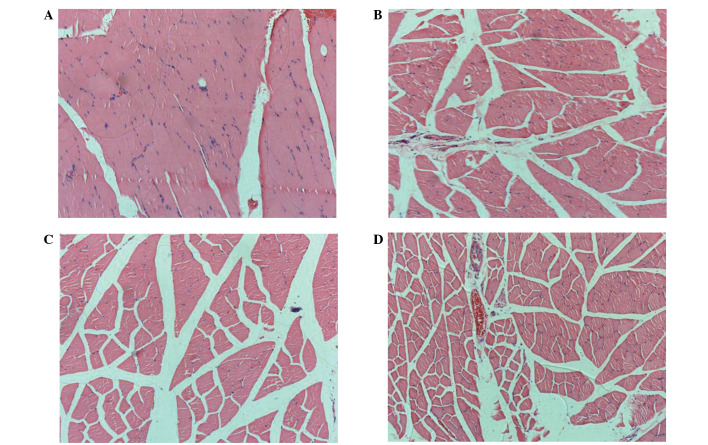
Haematoxylin and eosin-stained sections of the gastrocnemius muscle: Implantation group at (A) 3 and (B) 6 weeks; and control group at (C) 3 and (D) 6 weeks. Magnification, ×3000.

At three weeks, the muscles of the control group exhibited the characteristic features of muscle fibre atrophy, including fibrosis and fatty deposits. The changes were more evident at six weeks (Figs. 5C and D). In comparison with the control group, the electrical stimulation led to a marked improvement in the morphology of the muscles from three to six weeks. Reductions in the endomysial space and in the thickness of the perimysium were observed, and the muscle fibres appeared larger and more tightly packed within the fascicles (Figs. 5A and B). These changes were reflected in the results of the morphometric analysis (Table III). The CSA of the gastrocnemius muscles and the total number of muscle fibers were demonstrated to differ significantly between the implantation and control groups at the three- and six-week time points (P<0.05 for each). These differences were due to the atrophy of the muscles in control group.

#### Transmission electron microscopy

**Figure 6.**
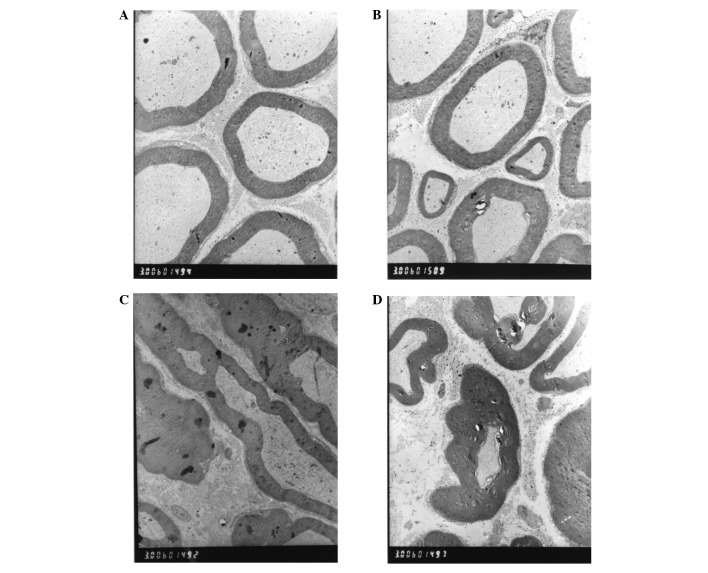
Transmission electron microscopy of the sciatic nerve: Implantation group at (A) 3 and (B) 6 weeks; and control group at (C) 3 and (D) 6 weeks. Magnification, ×3000.

Transmission electron microscopy of the control group, performed three weeks following the surgery, revealed that the distal axonal portion of the crushed nerve exhibited microfibrillar breakdown, swollen chondriosomes and myelin irregularities (Fig. 6C). Six weeks following the surgery, the breakdown of the microfibrils and microtubules in the control group still existed, and the chondriosomes exhibited swelling (Fig. 6D). With regard to the implantation group, from three to six weeks following the surgery, extensive Schwann cell proliferation and immature myelin formation were observed in the sciatic nerve (Figs. 6A and B).

## Discussion

Peripheral nerve injuries are common and have a marked impact on the everyday life of the general population. The restoration of function following a peripheral nerve injury continues to present a significant challenge. Although in the last century there has been an increase in the understanding of peripheral nerve rehabilitation (18–21), there remains a lack of totally effective treatment methods, and a fully functional outcome, particularly of motor function, is rarely achieved. However, non-surgical approaches, such as FES, have been developed to enhance nerve recovery, and, at present, FES is widely used to improve the rehabilitation of patients with neural impairments (22). A variety of electrical stimulation techniques have been developed, including transcutaneous electric nerve stimulation (TENS) (23,24), spinal cord stimulation (SCS) (25,26) and deep brain stimulation (DBS) (14,27). However, there remain disadvantages with the use of these procedures, for example none of the procedures have exhibited efficacy in the treatment of peripheral nerve injury, and the use of certain treatments, such as TENS is limited, particularly in cases that require the application of electrical stimulation for an extended period of time. Therefore, there is a requirement for further fundamental studies.

The development of medical implantable microsystems has led to a number of potential opportunities for the treatment of peripheral nerve injuries, particulary as these implants enable the establishment of a man-machine interface with the peripheral nervous system. There have recently been investigations into a variety of techniques for contacting nerves at different anatomical levels (23–27). In the present study, we developed an implantable electrical stimulator that incorporated the features that were considered essential in an ideal device. These requirements included the stimulator being suitable for a broad range of applications, including continuous stimulation for prolonged periods, and being programmable, with easily adaptable parameters. In addition, there were requirements for controlled-current sources, a small size and a low power consumption.

Following the methods described by Beer *et al* (28) and Varejão *et al* (29), we used Sprague-Dawley rats to establish an animal model of peripheral nerve injury, and then used this model to evaluate the previously mentioned requirements of the implantable electrical stimulator. Following the implantation of the electrical stimulators into the rats, the device was demonstrated to be reliable and stable for the duration of the experiment (≤6 weeks); none of the implanted devices failed. The ethological and macroscopic evaluations of the model animals revealed that the stimulator met the requirement of biocompatibility. Pathological examination of the rats that did not receive electrical stimulation demonstrated that the crushed nerve exhibited various degrees of Wallerian degeneration in the 3–6-week period subsequent to the crush injury. However, in the animals that received daily *in vivo* electrical stimulation, transmission electron microscopy revealed Schwann cell proliferation and immature myelin formation, in the same 3–6-week period. This result confirmed the success of the establishment of the peripheral nerve injury model, in addition to revealing that the selected stimulation pattern (bipolar pulses with a duration of 400 *μ*sec per phase, an amplitude of 0.8 V and a frequency of 60 Hz) was sufficient and functional. One of the critical factors in the electrical stimulation technique is the stimulation parameters that are used. It has been observed that, for short-term electrical stimulation, a frequency of 20 Hz is effective for the regeneration of a transected nerve (30). However, for long-term electrical stimulation, a frequency of 100 Hz was reported to have an enhanced regenerative effect, in comparison with that of 20 Hz (31). Another important factor in electrical stimulation is the type of stimulation waveform. A biphasic current pulse has previously been used in the electrical stimulation of cells and tissues, and has been demonstrated to produce safe and effective results (32). In the present study, the stimulation parameters were selected from the results of our preliminary experiments, and were revealed to be effective with regard to peripheral nerve rehabilitation in rats. The results of the study demonstrated the presence of muscle atrophy in the control group, a phenomenon that, clinically, is a secondary occurence to the peripheral nerve injury. Electrical stimulation may be used in the treatment of this disorder. In the rats in the implantation group, the gastrocnemius muscle was revealed to have recovered by the 6-week time point, which indicated that the electrical stimulation was critical to the recovery. The study, therefore, demonstrated that the implantable electrical stimulator was able to prevent denervated muscles from undergoing atrophy, in addition to accelerating the regeneration of the nerve.

There was, however, a clear disadvantage to the implantable stimulator, in that there was a lack of direct access to the electrode waveforms. To resolve this, it may be possible to add light emitting diodes to the circuit, to provide confirmation of the delivery of a current pulse to the bipolar electrodes. A future development of the implantable stimulators may be to include microsystem stimulation parameters that may be adapted during the different periods of recovery of the injured peripheral nerve, and which may be communicated via wireless techniques. In addition, there is a requirement for investigations into the development of devices with variable current amplitude outputs and multichannel stimulation capabilities.

In the current study, we designed an effective implantable electrical stimulator that was suitable for implantation in an rat model of peripheral nerve injury, and then analyzed its use in the electrical stimulation of the peripheral nerve. With simple modifications to the stimulation parameters, this stimulator may have clinical applications in numerous other areas of neuroscience.

## Figures and Tables

**Table I.** Sciatic nerve function index (SFI) in the implantation and control groups.

Week	SFI (%)	
	Implantation	Control
3	−16.27±3.01^a^	−40.50±3.81
6	−11.16±1.90^a^	−51.98±5.35

Results are expressed as the mean ± standard deviation.

a P<0.05 compared with the control group.

**Table II.** Recovery rate of the motor nerve conduction velocity in the implantation and control groups.

Week	Recovery rate (%)	
	Implantation	Control
3	44.8±3.0^a^	22.7±2.4
6	52.5±1.5^a^	23.2±2.2

Results are expressed as the mean ± standard deviation.

a P<0.05 compared with the control group.

**Table III.** Complete cross-sectional area (CSA) of muscles and the total number of muscle fibers in the implantation and control groups.

Weeks	CSA (mm^2^)		Number of fibers	
	Implantation	Control	Implantation	Control
3	52.4±3.5^a^	37.8±2.6	13685±1024^a^	11087±1128
6	66.9±4.7^a^	31.3±1.8	15373±1198^a^	9872±1027

Results are expressed as the mean ± standard deviation.

a P<0.05 compared with the control group.
